# Correlation between *in vitro* fertilization and artificial insemination in Holstein bulls

**DOI:** 10.5713/ab.20.0665

**Published:** 2021-02-16

**Authors:** Wei Sun, Yunxia Li, Jie Su, Xiangnan Bao, Rui Ding, Gaoping Zhao, Guifang Cao, Shuxiang Hu, Jianguo Wang, Qingyuan Sun, Haiquan Yu, Xihe Li

**Affiliations:** 1 College of Life Science, Inner Mongolia University, Hohhot 010020, China; 2 The State Key Laboratory of Reproductive Regulation and Breeding of Grassland Livestock, Inner Mongolia University, Hohhot 010020, China; 3 Inner Mongolia Saikexing Institute of Breeding and Reproductive Biotechnology in Domestic Animal, Hohhot 011517, China; 4 College of Veterinary Medicine, Inner Mongolia Agricultural University, Hohhot 010018, China; 5 Institute of Zoology Chinese Academy of Science, Beijing 100101, China

**Keywords:** Artificial Insemination (AI), Correlation Analysis, Holstein Bull, *In vitro* Fertilization (IVF)

## Abstract

**Objective:**

Owing to the lack of a breeding index for efficient and quick fertility evaluations of Holstein bulls when using traditional or genome-wide detection methods, this study aimed to determine whether *in vitro* fertilization (IVF) could be used as an indicator of conception rate of artificial insemination (AI).

**Methods:**

Conventional and sexed frozen semen from nine bulls were used for IVF and AI.

**Results:**

The IVF and AI conception rates of each bull were confirmed to be positively correlated between the conventional frozen and sexed frozen semen. The correlation coefficient R values of nine bulls between IVF and AI methods were 0.73 and 0.97 for the conventional frozen and sexed frozen semen, respectively. The average conception rate of three bulls undergoing AI was 69.5% and 64.2%, 61.8% and 58.8%, and 48.2% and 46.2% in first-, second-, and third-born cows when conventional frozen and sexed frozen semen were used, respectively, which showed a positive correlation with the fertilization rate in the same parity. We propose an evaluation standard to assess the fertilization ability of bulls based on their IVF test results, which is categorized into three grades: grade one, normal fertility bull with an AI conception rate of 40%±5% and IVF rate of 45% to 60%; grade two, higher fertility bull with an AI conception rate of 50%±5% and IVF rate of 61% to 80%; and grade three, highest fertility bull with an AI conception rate of 60%±5% and IVF rate of >80%.

**Conclusion:**

These findings reveal that IVF results can be used as a breeding index for bulls to evaluate their AI conception ability, which may shorten the time required to select bulls for breeding.

## INTRODUCTION

Several factors, including bull semen quality, feeding management, health status, and insemination technology, affect the artificial insemination (AI) conception rate in Holstein cows [1–5]. In contrast to AI, *in vitro* fertilization (IVF) can be performed under consistent laboratory conditions to complete the fertilization process and early embryo development [6–8]. The conventional method for evaluating the fertilization ability of bulls is the AI test, but it is time-consuming, requires approximately 4 to 5 years, and is expensive; therefore, it is necessary to develop a simple and accurate method for evaluating the AI fertilization ability of bulls [9].

AI and IVF are widely used treatments for assisted reproduction. AI is the main breeding technology for large-scale cattle breeding, which has been promoted by sexed semen technology in recent years [10,11]. Improving the fertilization ability of bulls is one of the key measures for increasing the conception rate of AI. IVF is widely used in the production and introduction of livestock embryos [12] and treatment of human infertility. In addition, IVF is a commonly used technique for testing fertilization ability of male animals [13]. The combination of these two technologies will benefit the breeding industry. A previous study [14] performed IVF to investigate validity of IVF in evaluating AI bull fertility. However, to date, there has been no detailed investigation on the relationship between AI and IVF in sexed frozen semen, which could provide a new index for evaluating bull fertility. In this study, after using either conventional frozen semen or sexed frozen semen, the correlations between IVF and AI conception rates in nine bulls with different breeding abilities were analyzed. The results of this study may provide a new evaluation index for Holstein bull breeding ability and guarantee the efficiency of the industrial application of AI.

## MATERIALS AND METHODS

The Institutional Animal Care and Use Committee of Inner Mongolia University approved the experimental protocol employed in this study (SYXK2014-0002).

### Reagents

All reagents were purchased from Sigma-Aldrich Co (St. Louis, MO, USA), unless indicated otherwise.

### Production of conventional frozen and sexed frozen semen

**Figure 1:**
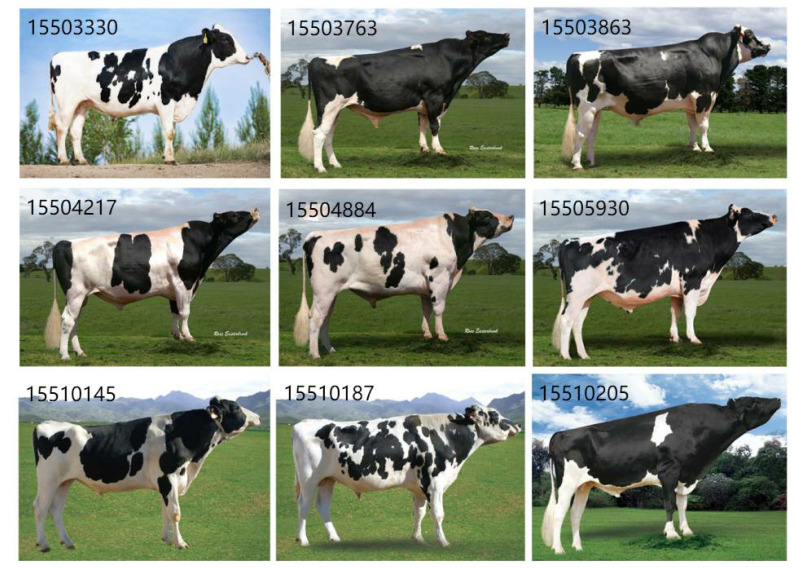
Nine bulls used for this experiment. The number in the upper left of each bull is bull number registered by Holstein Association of China.

Nine Holstein bulls aged 3 to 5 years were randomly selected from the bull station of Inner Mongolia Saikexing Reproductive Biotechnology (Hohhot, China) Co., Ltd. The registration numbers of the bulls are provided in Figure 1. Conventional frozen and X-sexed frozen semen were prepared using the straw freezing procedure. Populations of spermatozoa for conventional and X-sexed frozen semen were 20 million and 2 million, respectively, in each 0.25 mL straw [6].

### *In vitro* fertilization

Ovaries were obtained from cows at a local slaughterhouse and were transported to the laboratory at 35°C in 0.9% NaCl containing 70 mg/mL kanamycin. Cumulus-oocyte complexes (COCs) were aspirated from medium-sized follicles (approximately 5 mm in diameter) with a 10-mL disposable syringe. Only COCs surrounded by a compact cumulus mass with an evenly granulated cytoplasm were harvested and then washed thrice in a maturation medium. A total of 60 to 80 COCs were transferred into each well of a Nunc 4-well dish containing 500 μL pre-equilibrated maturation medium (TCM199) [15–17] at 38.5°C in 5% CO_2_ and 100% humidity. Bull spermatozoa were thawed in a water bath set at 38°C and washed twice in Brackett and Oliphant (BO) medium [18]. Centrifugation was performed at 1,680×g for 5 min in 10 mL BO medium. The final sperm pellet was allowed to settle at 37°C for 90 s without shaking, and the supernatant was collected using the swim-up method. Motility was assessed by placing a drop of semen (5 mL) on a prewarmed slide and assessing 100 sperms under a phase contrast microscope (40). A minimum of three straws from each of the three ejaculates from each bull (n = 9 straws per bull) were analyzed for a range of *in vitro* sperm functional assessments [9,14]. BO’s fertilization medium [18] was placed as 50 μL drops covered with warm mineral oil in a 35-mm culture dish and incubated at 38.5°C in 5% CO_2_. The COCs that matured *in vitro* were washed thrice and placed in 50 μL drops of pre-equilibrated IVF medium covered with warm mineral oil in a 35-mm culture dish (30 COCs per drop). Fifty tiny drops of supernatant solution containing sperm (minimum concentration of 1.0×10^7^ spermatozoa/mL) were then added to the drops that contained COCs and were incubated at 38.5°C under conditions of 5% CO_2_ and high humidity for fertilization [17–19].

At 6 h after coculturing, COCs were washed and transferred (30 to 50 COCs per well) to a Nunc 4-well dish containing 500 μL embryo culture medium [9] covered with 500 μL mineral oil and cultured in modular incubation chambers (615300; ICN Biomedical, Inc., Aurora, OH, USA) at 38.5°C in air containing 5% CO_2_ and 100% humidity for 48 h to assess fertilization as indicated by embryo cleavage. Cleaved 2-cell embryos were continuously cultured for 7 days to observe blastocyst formation [20]. The sex of the blastocysts was analyzed by polymerase chain reaction [21]. For each bull, three different batches of frozen semen were used for the IVF experiments.

### Artificial insemination

Heifers aged 13 to 15 months, weighing approximately 350 kg, and cows with 2 to 3 parity with a regular estrus period of 60 to 90 days after delivery were selected for AI following healthy examination, and also we avoid normally hot summer of July to August and cold winter of December to January, to eliminate the differences due to the climate influence. Then, AI with conventional frozen or sexed frozen semen was performed in three 5,000 large-scale Holstein farms. A pedometer computer information monitoring system was used to confirm the estrus and ovulation periods of the cow. The ovulatory side was determined by rectal examination, and the uterine horn was injected with semen (2×10^7^ sperm) 10 to 12 h after the estrus period (4 to 6 h before or after ovulation). After 35 days of AI, B ultrasonic detection was used to confirm the conception of the cows. AI treatment for all recipients of 1 to 3 parities were performed by same AI and veterinary working group.

### Statistical analysis

The AI conception and IVF rates of conventional frozen and sexed frozen semen from different bulls were analyzed using the SPSS17.0 software [22]. Nine bulls were classified according to the results of the experiment. Data are presented as per the results of X^2^ analysis.

## RESULTS

### Assessment of sperm quality

Semen quality indices, including sperm density, motility, and deformity rates, of nine bulls used in the experiment are presented in Table 1. The sperm density of each bull was >700 million/mL; average sperm motility was >60%, and the average sperm deformity rate was <18%. The quality of sperm conforms to the national standard for the production of frozen semen.

### Correlation between the *in vitro* fertilization and artificial insemination conception rates for conventional frozen semen

**Figure 2:**
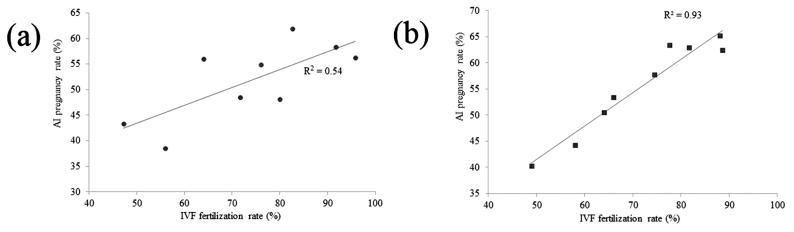
Correlation between the AI pregnancy rate and IVF fertilization rate by using conventional frozen semen (a) and sexed frozen semen (b). Correlation cofficients between AI pregnancy rate and IVF fertilization rate by using (a) conventional frozen semen (r = 0.73, p<0.05) and (b) sexed frozen semen (r = 0.97, p<0.01) were shown. Two lines show the trends in the data. IVF, *in vitro* fertilization; AI, artificial insemination.

A total of 491 mature oocytes were used for IVF, and 8,800 cows were used for AI, with conventional frozen semen from nine bulls. The rates of fertilization and blastocyst development, and embryonic sex of IVF as well as the rates of conception and calf sex of AI are summarized in Table 2. Three different batches of conventional frozen semen of each bull were used for IVF experiments, and the corresponding AI results were obtained. The rates of fertilization, blastocyst development, and female embryos in the IVF experiments ranged from 48.4% to 95.8%, 29.5% to 37.2%, and 44.4% to 58.8% and the rates of AI conception and female calves ranged from 38.5% to 61.9% and 47.8% to 51.0% from the nine bulls, respectively. The IVF rates of three bulls (15504884, 15503863, and 15503763) were >81.4%, whereas counterpart AI conception rates ranged from 56.2% to 61.9%. For four bulls (15504217, 15510145, 15503330, and 15510205), IVF rates ranged from 64.0% to 80.0% and the counterpart AI conception rates ranged from 48.1% to 56.0%. For two bulls (15510187 and 15505930), IVF rates ranged from 48.4% to 56.0% and the counterpart AI conception rates ranged from 38.5% to 43.3%. A correlation analysis showed that there was a positive correlation between the AI conception and IVF rates (r = 0.73, p<0.05, Figure 2a).

### Correlation between the *in vitro* fertilization and artificial insemination conception rates of sexed frozen semen

A total of 686 mature oocytes were used for IVF and 7,750 cows were used for AI, with sexed frozen semen from the same nine bulls. The rates of fertilization, blastocyst development, and female embryos of IVF and the rates of conception and female calves of AI are summarized in Table 3. Three different batches of sexed frozen semen of each bull were used for the IVF experiments, and the corresponding AI results were obtained. The rates of fertilization, blastocyst development, and female embryos ranged from 48.9% to 88.6%, 27.1% to 39.0%, and 75.0% to 100.0%, and the rates of AI conception and female calves ranged from 40.3% to 65.2% and 90.0% to 95.0% from the nine bulls, respectively. IVF rates of three bulls (15504884, 15503863, and 15503763) was >81.7%, and counterpart AI conception rates ranged from 62.4% to 65.2%. For four bulls (15504217, 15510145, 15503330, and 15510205), IVF rates ranged from 64.0% to 77.6% and counterpart AI conception rates ranged from 50.5% to 63.4%. For two bulls (15510187 and 15505930), IVF rates ranged from 48.9% to 58.0% and counterpart AI conception rates ranged from 40.3% to 44.3%. A correlation analysis showed that there was a significant correlation between the AI conception and IVF rates (r = 0.97, p<0.01, Figure 2b).

### Effects of artificial insemination cow parities on artificial insemination conception rate

Three bulls (15504884, 15503863, and 15503763) with high IVF rates were selected to produce conventional frozen and sexed frozen semen on a large scale. AI conception rates of 23,610 cows with different cow parities were compared (Table 4). The average conception rate of AI was 69.5% in first-born cows, 61.8% in second-born cows, and 48.2% in third-born cows when conventional frozen semen was used, whereas it was 64.2%, 58.8%, and 46.2%, respectively, when sexed frozen semen was used. Compared with the average fertilization rate of IVF, the AI conception rate of both conventional frozen and sexed frozen semen decreased as the AI cow parity increased; however, a correlation with the fertilization rate was noted (p<0.05).

The software SPSS17.0 was used to analyze the variance and to test the significant difference and correlation between the IVF and AI conception rates of the bulls (Table 5). Consequently, the AI conception rate showed a significant correlation with the IVF rate (p<0.05).

## DISCUSSION

### *In vitro* fertilization was positively correlated with artificial insemination conception rates

Some studies suggest that the outcome of IVF is useful for predicting the AI conception rate of bull semen and the ability to support early embryo development [14,23]; however, there are some controversies that need to be investigated further [9,24], and also take an evaluate standard from IVF to AI conception rate (Table 6).

In this study, the correlation between the AI conception and IVF rates were investigated by using conventional and sexed frozen semen from nine bulls. The average rates of IVF and AI conception were approximately 48.4% to 95.8% and 43.3% to 58.4%, respectively, when using conventional frozen semen. The average rates of IVF and AI conception were approximately 48.9% to 88.6% and 40.3% to 65.2% respectively when using sexed frozen semen. Based on these results, we found that the IVF rates, which were compartmentalized with different fertility abilities, were positively correlated with AI conception rates in each of the nine bulls by using either conventional frozen semen (r = 0.73, p<0.05) or sexed frozen semen (r = 0.97, p<0.01), including AI recipient changes with different AI recipient parities.

### New evaluation standard for the fertilization ability of bulls based on *in vitro* fertilization test results

Considering the rates of IVF and embryonic development and the sex ratio, we conclude that the IVF rate in both conventional and sexed frozen semen can be used as a criterion for evaluating the bull AI conception ability. Thus, we propose an evaluation standard for the fertilization ability of bulls as per their IVF test results. This evaluation standard of the bull AI conception ability is categorized into three grades (Table 6): grade one, normal fertility bulls with an AI conception rate of 40%±5% and IVF rate of 45% to 60%; grade two, higher fertility bulls with an AI conception rate of 50% ±5% and IVF rate of 61% to 80%; and grade three, highest fertility bulls with an AI conception rate of 6%±5% and IVF rate of >80%.

## CONCLUSION

The IVF can be used as a bull breeding index to evaluate bull AI conception, which was classified into three grades, namely, normal fertility, higher fertility, and highest fertility. It provides a more useful index than the genome-wide detection of Holstein bull breeding ability.

## Figures and Tables

**Table 1:** Sperm quality of nine bulls

Sequence number	Bull number	CPI	Sperm density (10^9^/mL)	Sperm motility %	Sperm deformity rate %
1	15510187	202	15.8	65	13.3
2	15505930	1,505	7.6	61	14.0
3	15504217	893	17.3	69	13.1
4	15510145	288	10.5	67	14.2
5	15503330	405	14.0	66	15.0
6	15510205	135	13.2	63	13.7
7	15504884	172	18.1	63	14.5
8	15503863	−986	12.0	67	12.1
9	15503763	−332	13.7	65	14.4

CPI, China performance index.

**Table 2:** Comparison of the *in vitro* fertilization and artificial insemination conception rates of conventional frozen semen from nine bulls

Bull number	IVF rates^1)^			AI rates^1)^	
	
	2-Cell %	Blast. %	Embryos ♀/Total %2)	Conception %	Calves ♀/Total %2)
15510187	48.4 (31/64)	35.5 (11/31)	45.5 (5/11)	43.3 (265/612)	49.1 (130/265)
15505930	56.0 (28/50)	32.1 (9/28)	44.4 (4/9)	38.5 (279/725)	47.8 (133/278)
15504217	64.9 (37/57)	32.4 (12/37)	58.3 (7/12)	56.0 (1,100/1,963)	48.4 (52/110)
15510145	71.7 (43/60)	37.2 (16/43)	50.0 (8/16)	48.5 (204/421)	51.0 (53/104)
15503330	74.0 (37/50)	35.1 (13/37)	46.2 (6/13)	54.9 (388/707)	47.9 (186/388)
15510205	80.0 (44/55)	29.5 (13/44)	53.8 (7/13)	48.1 (206/428)	50.5 (104/206)
15504884	81.4 (48/59)	35.4 (17/48)	58.8 (10/17)	61.9 (1,184/1,912)	49.8 (590/1,184)
15503863	91.7 (44/48)	36.4 (16/44)	56.3 (9/16)	58.4 (976/1,671)	51.0 (498/976)
15503763	95.8 (46/48)	37.0 (17/46)	53.0 (9/17)	56.2 (203/361)	50.2 (102/203)

IVF, *in vitro* fertilization; AI, artificial insemination.

1) The rates of 2-cell and blastocyst embryos were calculated at 48 h and 7 days after IVF, respectively.

2) The rates of ♀ embryos/total of IVF blastocyst embryos were analyzed following polymerase chain reaction, and each bull was tested but not for >50 embryos.

**Table 3:** Comparison of the *in vitro* fertilization and artificial insemination conception rates of sexed frozen semen from nine bulls

Bull number	IVF rates^1)^			AI rates^1)^	
	
	2-Cell %	Blast. %	Embryos ♀/Total %2)	Conception %	Calves ♀/Total %2)
15510187	48.9 (22/45)	31.8 (7/22)	100.0 (7/7)	40.3 (106/263)	92.5 (98/106)
15505930	58.0 (87/150)	32.2 (28/87)	93.7 (15/16)	44.3 (144/325)	94.4 (136/144)
15504217	64.0 (48/75)	27.1 (13/48)	92.3 (12/13)	50.5 (220/436)	90.0 (198/220)
15510145	66.0 (33/50)	36.4 (12/33)	75.0 (9/12)	53.4 (327/612)	92.0 (301/327)
15503330	74.5 (41/55)	39.0 (16/41)	87.5 (14/16)	57.7 (329/570)	94.8 (312/329)
15510205	77.6 (45/58)	31.1 (14/45)	100.0 (14/14)	63.4 (398/628)	91.9 (366/398)
15504884	88.0 (73/83)	35.6 (26/73)	94.4 (17/18)	65.2 (922/1,414)	93.0 (857/922)
15503863	81.7 (67/82)	31.3 (21/67)	100.0 (16/16)	62.9 (1,331/2,116)	91.0 (1,211/1,331)
15503763	88.6 (78/88)	34.6 (27/78)	88.2 (15/17)	62.4 (865/1,386)	95.0 (822/865)

IVF, *in vitro* fertilization; AI, artificial insemination.

1) The rates of 2-cell and blastocyst embryos were calculated at 48 h and 7 days after IVF, respectively.

2) The rates of ♀ /total of IVF blastocyst embryos were analyzed following polymerase chain reaction, and each bull was tested but not for >50 embryos.

**Table 4:** Comparison of the conception rate in different artificial insemination cow parities

Item	1 parity^1)^	2 parity^1)^	3 parity^1)^
Conventional frozen semen AI %	69.5 (2,677/3,852)	61.8 (1,853/2,998)	48.2 (1,915/3,974)
Sexed frozen semen AI %	64.2 (2,606/4,059)	58.8 (2,430/4,132)	46.2 (2,123/4,595)

AI, artificial insemination.

1) The average IVF rates of bulls 15504884, 15503863, and 15503763 in conventional and sexed frozen semen were 90.0% and 86.1%, respectively, which were used in the above AI treatment with 1 to 3 parities.

**Table 5:** Correlation between the artificial insemination conception and *in vitro* fertilization rates using conventional and sexed frozen semen

Items	Index
Conventional frozen semen	0.925*
Sexed frozen semen	0.963**

* Indicates difference p<0.05;

** Indicates difference p<0.01.

**Table 6:** Grading the standard to evaluate the conception ability of bulls

Standard for evaluation of fertility in bulls	IVF rate range (%)	AI conception rate range (%)	Bull number
Grade 1: normal fertility bulls	45 to 60	40±5	15505930
			15510187
Grade 2: higher fertility bulls	61 to 80	50±5	15504217
			15510145
			15503330
			15510205
Grade 3: highest fertility bulls	>80	60±5	15504884
			15503763
			15503863

IVF, *in vitro* fertilization; AI, artificial insemination.
